# Statistical Methods for the Investigation of Solvolysis Mechanisms Illustrated by the Chlorides of the Carbomethoxy Protecting Groups NVOC and FMOC

**DOI:** 10.1155/2015/941638

**Published:** 2015-02-12

**Authors:** Malcolm J. D'Souza, Jasbir K. Deol, Maryeah T. Pavey, Dennis N. Kevill

**Affiliations:** ^1^Department of Chemistry, Wesley College, 120 N. State Street, Dover, DE 19901-3875, USA; ^2^Department of Chemistry and Biochemistry, Northern Illinois University, DeKalb, IL 60115-2862, USA

## Abstract

The solvolysis of 4,5-dimethoxy-2-nitrobenzyl chloroformate (NVOC-Cl, **1**) is followed at 25.0°C in twenty hydroxylic solvents. A comparison with previously published rates for benzyl chloroformate and *p*-nitrobenzyl chloroformate indicates that the inductive effect of the nitro and the two methoxy groups strongly influences the rate of reaction. For **1**, the specific rates of solvolysis are correlated using an extended Grunwald-Winstein (G-W) treatment. A direct comparison with the data for phenyl chloroformate (PhOCOCl) in identical solvents strongly suggests that the addition step within an addition-elimination mechanism is rate-determining for both substrates. A reevaluation of the kinetic data for 9-fluorenylmethyl chloroformate (FMOC-Cl, **2**) involves a correlation of log⁡(*k*/*k*_*o*_)_2_ versus log⁡(*k*/*k*_*o*_)_PhOCOCl_. In this plot, deviations were observed in solvents rich in a hydrogen-bonding fluoroalcohol component. Omitting the aqueous fluoroalcohol rate measurements for **2** in an analysis using the extended G-W equation suggested the occurrence of dual pathways differing in the dependences upon the ionizing power and nucleophilicity of the solvent. In addition, the fluorenyl ring is rotated out of the plane containing the ether oxygen and, as a result, PhOCOCl is found to solvolyze 20 times faster than **2** in ethanol and methanol.

## 1. Introduction

**Figure 1:**
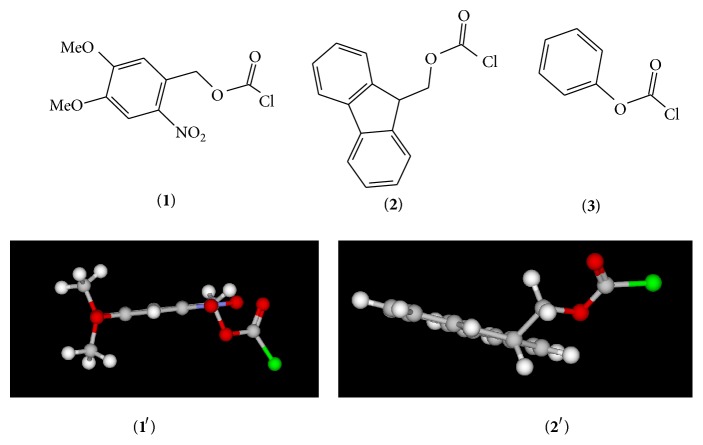
Molecular structures of 4,5-dimethoxy-2-nitrobenzyl chloroformate (NVOC-Cl,** 1**), 9-fluorenylmethyl chloroformate (FMOC-Cl,** 2**), and phenyl chloroformate (PhOCOCl,** 3**). The 3D structures for 4,5-dimethoxy-2-nitrobenzyl chloroformate (**1**′) and 9-fluorenylmethyl chloroformate (**2**′).

6-Nitroveratryl chloroformate is the synonym for 4,5-dimethoxy-2-nitrobenzyl chloroformate (NVOC-Cl,** 1**). Due to its chemical stability and ease of removal, this chloroformate ester is used to introduce the 6-nitroveratryl group (NVOC protecting group) in a variety of amino acid synthetic applications [1]. Similarly, 9-fluorenylmethyl chloroformate (FMOC-Cl,** 2**) is used to introduce the fluorenylmethyloxycarbonyl (FMOC) group to give the FMOC carbamate in solid and solution phase peptide synthetic processes [1]. In addition, both groups are incorporated in different methods used for synthesizing diverse polymer sequences for agricultural products [2]. The molecular structures for NVOC-Cl (**1**) and FMOC-Cl (**2**) and their corresponding 3D images (**1**′) and (**2**′) are shown in Figure 1. Details of the commercial 3D image rendering program are provided in Section 4.

**Scheme 1:**
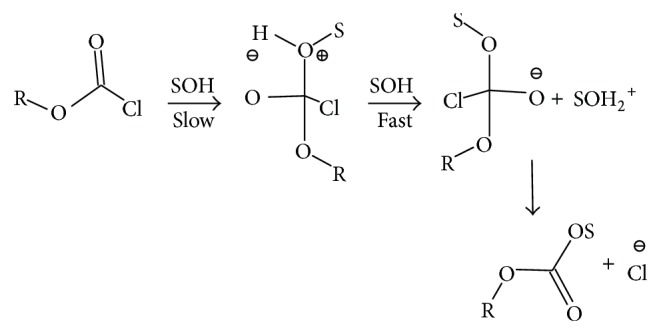
Stepwise addition-elimination mechanism through a tetrahedral intermediate for chloroformate esters.

Chloroformates (ROCOCl) in general are important organic building blocks [1–3] employed in the chemical industry due to reduced reactivity when compared to the rapidly reacting acid chlorides of the RCOCl type. The slower rates of reaction are a result of the alkoxycarbonyl (or aryloxycarbonyl) resonance stabilization [3]. Several groups [4–13] have extensively examined the mechanism of solvolysis of phenyl chloroformate (PhOCOCl,** 3**) in a very wide range of solvents. For PhOCOCl, in 49 solvents, a two-step addition-elimination (association-dissociation) process (Scheme 1) was believed [6, 11] to prevail.

The Grunwald-Winstein (G-W) equations ((1) and (2)) [14, 15] are linear free energy relationships (LFERs) used to quantify influences of solvent effects on a given substrate. In (1) and (2), *k* is the specific rates of solvolysis in a given solvent, *k*_*o*_ is the specific rate in 80% aqueous ethanol (an arbitrarily fixed standard solvent), and *c* is a constant residual term. In both equations [14, 15], *m* is a measure of the sensitivity to changes in solvent ionizing power *Y*. In (2) [15], *l* is a measure of the sensitivity to changes in solvent nucleophilicity *N*. Scales for both *N* [16, 17] and *Y* [18, 19] are established. An *N*_T_ [17] scale based on the solvolysis of S-methyldibenzothiophenium ion and a *Y*_Cl_ [18–20] scale based on the solvolysis of 1-adamantyl chloride are the preferred scales for the Grunwald-Winstein type analyses of chloroformate esters:

(1) $$\begin{array}{c} \log\left(\frac{k}{k_{o}}\right)=mY+c, \end{array}$$

(2) $$\begin{array}{c} \log\left(\frac{k}{k_{o}}\right)=lN+mY+c. \end{array}$$

Bentley [21–25] prefers the use of the one-term G-W equation (1) to analyze rate profiles. He also suggested [21–25] the use of similarity models (*Y*_sim_) to interpret dispersions observed when there is *π*-bond resonance stabilization adjacent to the reaction site. Alternatively, we prefer [26–32] modifying (1) and (2) to evaluate and explain the solvolysis of substrates with *π*-electron conjugation (including *α*-haloalkyl aryl compounds) at the *α*-carbon or in presence of intramolecular anchimeric assistance.

Recently, two review chapters have appeared [33, 34] showing the use of the G-W equations within studies of the solvolyses of haloformate esters and their thioanalogs. In these chapters, when using (2), we reemphasize [6, 11, 31, 33, 34] the use of the *l* value of 1.66 and *m* value of 0.56 (*l*/*m* ratio of 2.96) obtained for PhOCOCl (**3**) as an appropriate standard for a bimolecular carbonyl-addition pathway (Scheme 1) with a rate-determining addition step. We have shown [31, 33–36] that *l*/*m* values >2.7 are typical ratios for acyl halide solvolyses proceeding by an addition-elimination (A-E) pathway with the addition step being rate-determining.

In addition, for the solvolyses of octyl chloroformate and fluoroformate, we determined [35] the *k*_Cl_/*k*_F_ ratio to be somewhat below unity in mixtures of water with ethanol (EtOH), acetone, dioxane, or 2,2,2-trifluoroethanol (TFE). This is consistent with our initial proposal [6] of a rate-determining addition step in an addition-elimination process for haloformate esters. In solvents of very low nucleophilicity and very high ionizing power, an ionization mechanism was observed for some chloroformates [31, 33, 34]. We showed [31, 33, 34, 36] that the G-W *l*/*m* ratios between 0.5 and 1.0 are indicative of a unimolecular ionization (S_N_1) process with appreciable rear-side nucleophilic solvation, while values much smaller than 0.5 suggest the occurrence of an ionization-fragmentation process.

Like** 1** and** 2**, benzyl chloroformate (C_6_H_5_CH_2_OCOCl, CBZ-Cl) and* p*-nitrobenzyl chloroformate (*p*-NO_2_C_6_H_4_CH_2_OCOCl, PNZ-Cl) are chloroformate esters that are utilized in peptide synthesis [1, 2]. For CBZ-Cl in solvolysis, an *l*/*m* value of 0.38 was obtained in eleven fluoroalcohol-containing solvents and an *l*/*m* value of 3.42 was obtained in the remaining fifteen pure and aqueous-organic mixtures [37]. These ratios suggest a dichotomy of mechanism, with an ionization-fragmentation process accompanied by a loss of carbon dioxide occurring in the highly ionizing fluoroalcohol mixtures and an A-E process being dominant in the more nucleophilic solvents [37]. The presence of the solvolysis-decomposition (ionization-fragmentation) pathway for CBZ-Cl in the aqueous fluoroalcohols was confirmed by product studies showing varying amounts of the benzyl chloride decomposition product being formed [37]. For PNZ-Cl, the *l*/*m* ratio of 3.50 observed over the full range of solvent type was consistent with a carbonyl-addition (A-E) process [37, 38]. The *k*_MeOH_/*k*_MeOD_ ratio of 2.42 found [37, 38] for PNZ-Cl is a typical value for a carbonyl-addition pathway that is assisted by general-base catalysis [38].

Here we report on the specific rate constants obtained for NVOC-Cl (**1**) in twenty solvents of widely varying nucleophilicity (*N*_T_) and ionizing power values (*Y*_Cl_). We statistically analyze and report on the correlation values obtained for NVOC-Cl using the extended Grunwald-Winstein treatment (2). We compare the rate constants and the *l*/*m* ratio obtained (for NVOC-Cl) to the previously published data for CBZ-Cl [37] and PNZ-Cl [37, 38]. We also consider the resonance contributions from substituent effects [39] as a result of the presence of the nitro group and the two methoxy groups in NVOC-Cl.

Koh and Kang [40] completed a comprehensive evaluation using (2) of the rate profiles obtained for FMOC-Cl (**2**) in 33 aqueous-organic mixtures at 45.0°C. Omitting the TFE-EtOH mixtures in their calculations using (2), they obtained an *l* value of 0.95 and an *m* value of 0.39. They also observed a kinetic solvent isotope effect (*k*_MeOH_/*k*_MeOD_) ratio of 2.20. Basing their conclusions on their *l* and *m* values, they proposed that the solvolysis of** 2** proceeds through a bimolecular S_N_2 process [40].

Using (2), we reanalyze the Koh and Kang data [40] for FMOC-Cl (**2**) in all of the 33 solvents. Their reported *k*_MeOH_/*k*_MeOD_ value (2.20) [40] was close to the prior recorded KSIE ratio for PNZ-Cl (2.42) [37, 38] where a carbonyl-addition (A-E) process was definitively proposed. To gain further insights into the reactivity of NVOC-Cl (**1**) and FMOC-Cl (**2**), we employed Bentley's [21–25] similarity model approach and used the previously published log⁡(*k*/*k*_*o*_) values [6, 11] for PhOCOCl (**3**) solvolyses as the *Y*_sim_ scale.

## 2. Results and Discussion

In Table 1, we present the specific rates of solvolysis for 4,5-dimethoxy-2-nitrobenzyl chloroformate (NVOC-Cl,** 1**) in twenty binary aqueous-organic mixtures with varying *N*_T_ and *Y*_Cl_ values. The solvent mix includes the highly ionizing mixtures of aqueous fluoroalcohols where a unimolecular S_N_1-type (ionization) mechanism was proposed for several chloroformates [31, 33, 34].

In ethanol (EtOH), methanol (MeOH), and acetone, the specific rates of reaction for** 1** increase with an increase in water content in the solvent mixture. The rate constants also increase with the added water component in the aqueous 2,2,2-trifluoroethanol (TFE-H_2_O) and 1,1,1,3,3,3-hexafluoro-2-propanol (HFIP-H_2_O). In the TFE-EtOH mixtures, there is an increase in the rates of reaction as the proportion of ethanol is increased. The patterns of specific rates observed for the twenty solvents suggest that the nucleophilic component is critical in the transition-state structure.

A comparison of the pseudo-first order rates for NVOC-Cl (**1**), PNZ-Cl [37, 38], and CBZ-Cl [37] at 25.0°C reveals sequences where *k*_NVOC-Cl_ > *k*_PNZ-Cl_ > *k*_CBZ-Cl_ in the pure alcohols, in common binary mixtures of aqueous ethanol, methanol, and acetone and in the TFE-EtOH solvents. This rate trend indicates that the carbonyl-carbon reaction center in NVOC-Cl carries a much greater partial positive charge than the carbonyl reaction centers in PNZ-Cl and CBZ-Cl.

The 90% TFE is the only common aqueous fluoroalcohol in which the NVOC-Cl (**1**), PNZ-Cl, and CBZ-Cl are studied at 25.0°C and the rate trend is *k*_CBZ-Cl_ > *k*_NVOC-Cl_ > *k*_PNZ-Cl_. In the highly ionizing 90% HFIP, CBZ-Cl solvolyzes at a rate that is 177 times faster than** 1** and in 97% TFE, CBZ-Cl is 34 times faster than** 1**. For CBZ-Cl, an ionization-fragmentation reaction was previously proposed [37] in all of the aqueous fluoroalcohols.

**Figure 2:**
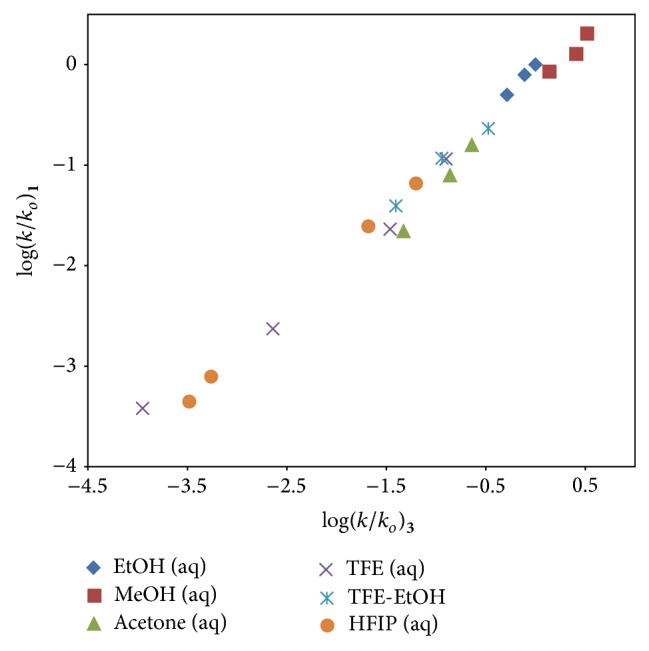
The plot of log⁡(*k*/*k*_*o*_) for 4,5-dimethoxy-2-nitrobenzyl chloroformate (**1**) at 25.0°C against log⁡(*k*/*k*_*o*_) for phenyl chloroformate (**3**) at 25.0°C in the twenty common pure and binary solvents studied.

Figure 2 shows a plot of log⁡(*k*/*k*_*o*_) for 4,5-dimethoxy-2-nitrobenzyl chloroformate (**1**) against log⁡(*k*/*k*_*o*_) for phenyl chloroformate (**3**) in the twenty common pure and binary solvents studied. The excellent correlation coefficient (*R* = 0.993) and *F*-test value (1316) are strong statistical indicators that a carbonyl-addition (A-E) process is also the dominant process for** 1** in all of the twenty solvents studied. The slope for this plot is 0.89 ± 0.02. The choice of** 3** as the standard is because the mechanism of solvolyses is well established [6, 11]. Its choice follows the rationale for choosing *p*-methoxybenzoyl chloride solvolyses as the standard [24] when unimolecular solvolyses of acyl chlorides (including chloroformate esters) are believed to be involved.

In Table 2, we report on the G-W analyses obtained for** 1** using (2) in all twenty solvents. For** 1**, we get an *l* value of 1.48 ± 0.13, an *m* value of 0.52 ± 0.08, *c* = 0.04 ± 0.11, *R* = 0.966, and an *F*-test value of 119. In the identical 20 solvents, a G-W analysis for** 3** results in *l* = 1.62 ± 0.14, *m* = 0.55 ± 0.09, *c* = 0.22 ± 0.13, *R* = 0.967, and *F*-test = 122. In these 20 solvents, the resulting *l*/*m* ratios for** 1** (2.85) and** 3** (2.95) show that the ratio for** 3** is marginally higher. This indicates that the mechanisms for** 1** and** 3** are essentially indistinguishable and that the tetrahedral transition states in a carbonyl-addition process are very similar.

**Figure 3:**
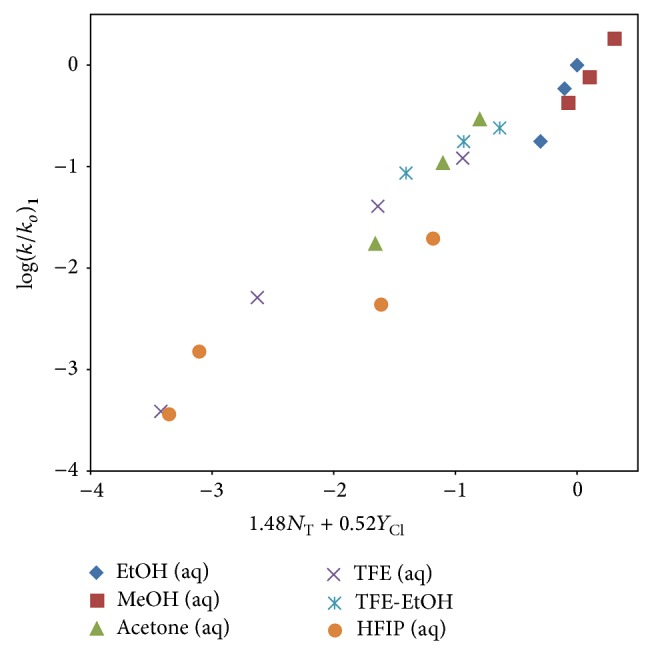
The plot of log⁡(*k*/*k*_*o*_) for 4,5-dimethoxy-2-nitrobenzyl chloroformate (**1**) against 1.48*N*_T_ + 0.52*Y*_Cl_ in the twenty pure and binary solvents studied.

A plot of log⁡(*k*/*k*_*o*_) for 4,5-dimethoxy-2-nitrobenzyl chloroformate (**1**) against 1.48*N*_T_ + 0.52*Y*_Cl_ in the twenty pure and binary solvents studied is shown in Figure 3. For use in this figure, we used (2), the previously published rate for** 3** [6, 11] in 80% EtOH, and an *l* value of 1.66 and *m* value of 0.56 [6, 11] to calculate a specific rate of 0.276 × 10^−5^ s^−1^ for solvolysis of** 3**, in 80% HFIP.

In Figure 1, the 3D image for 4,5-dimethoxy-2-nitrobenzyl chloroformate (**1**′) visibly shows that the two methoxy oxygens, the nitro group, and the aromatic ring are all very coplanar. As a result a greater inductive effect [39] is introduced in NVOC-Cl and therefore, in solvents where an addition-elimination process is proposed to be dominant, it solvolyzes much faster than PNZ-Cl and CBZ-Cl [37, 38].

In 30 solvents (without the TFE-EtOH mixture data points), Koh and Kang proposed [40] a bimolecular S_N_2 process for FMOC-Cl (**2**) on the basis of the magnitudes of the *l* (0.95) and *m* (0.39) values obtained.

Using (2) for all 33 solvents, we acquire an *l* value of 1.02 ± 0.08,  *m* = 0.44 ± 0.04, *c* = −0.04 ± 0.07, *R* = 0.925, and an *F*-test value of 89.

**Figure 4:**
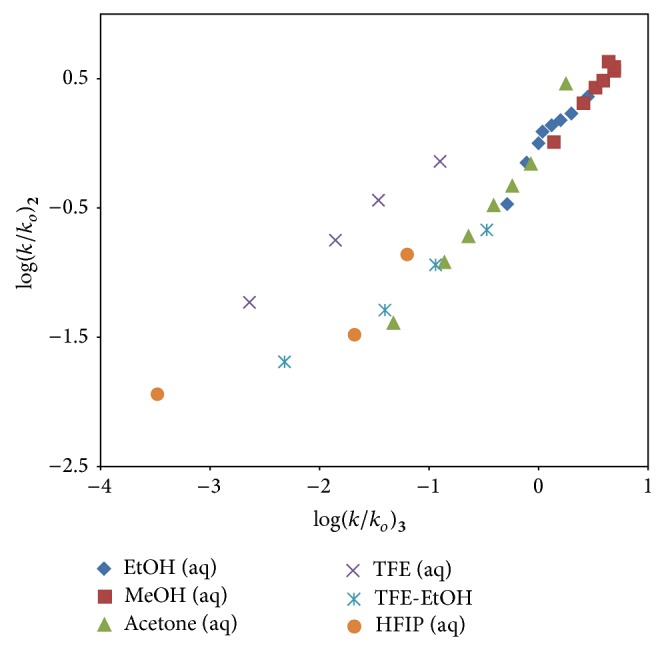
The plot of log⁡(*k*/*k*_*o*_) for 9-fluorenylmethyl chloroformate (**2**) against log⁡(*k*/*k*_*o*_) for phenyl chloroformate (**3**) in the thirty-three common pure and binary solvents studied.

Figure 4 shows the plot of the log⁡(*k*/*k*_*o*_) values for 9-fluorenylmethyl chloroformate (**2**) against the log⁡(*k*/*k*_*o*_) values for phenyl chloroformate (**3**) in all of the thirty-three common pure and binary solvents studied [40]. The correlation coefficient obtained for this plot is marginally acceptable with an *R* value of 0.924, *F*-test = 180, and slope = 0.64 ± 0.05. The graph (Figure 4) also shows the aqueous fluoroalcohols lying above the regression line. When this happens for the TFE-H_2_O and HFIP-H_2_O mixtures, earlier reports [11, 31, 33–36] on the solvolytic studies of chloroformate esters have indicated that a mechanistic shift occurs to one favoring an ionization process. Excluding the seven aqueous fluoroalcohol (TFE-H_2_O and HFIP-H_2_O) data points, the regression analysis for the remaining 26 solvents of log⁡(*k*/*k*_*o*_)_2_ versus log⁡(*k*/*k*_*o*_)_3_ results in a significantly improved correlation coefficient, *R* = 0.980, and *F*-test value = 588, with a slope of 0.88 ± 0.04. This linear association is robust and firmly indicates that, in these 26 solvents, the mechanism of reaction of** 2** is very similar to that observed for** 3**.

**Figure 5:**
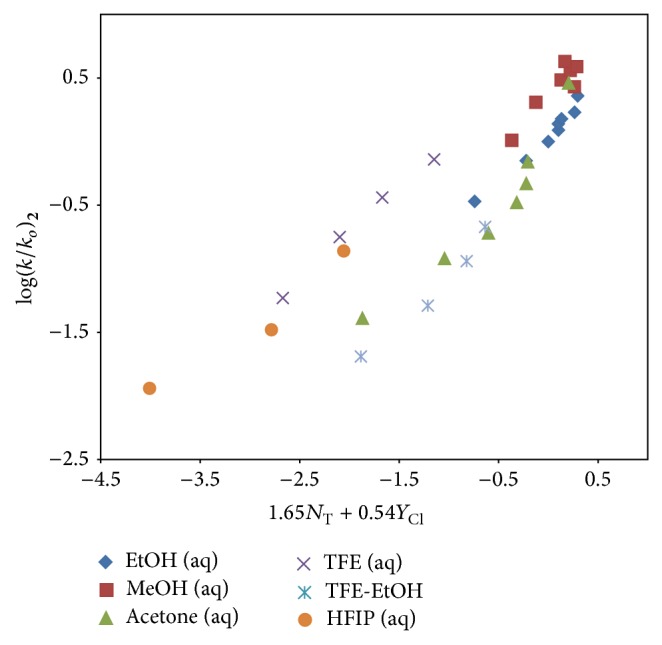
The plot of log⁡(*k*/*k*_*o*_) for 9-fluorenylmethyl chloroformate (**2**) against 1.65*N*_T_ + 0.54*Y*_Cl_ in the thirty-three pure and binary solvents studied. The points for TFE-H_2_O and HFIP-H_2_O are not included in the correlation. They are added to show the extent of their deviation from the correlation.

An analysis using (2) for solvolyses of** 2** in the 26 solvents (reported in Table 2) leads to values of *R* = 0.954, *l* = 1.65 ± 0.13, *m* = 0.54 ± 0.04, *c* = 0.13 ± 0.06, and *F*-test = 116. The *l*/*m* ratio of 3.04 is very similar to *l*/*m* ratios observed [11, 31, 33–36] for other acyl halide solvolyses which are believed to proceed by an addition-elimination (A-E) process with a rate-determining addition step. A plot of log⁡(*k*/*k*_*o*_)_2_ against 1.65*N*_T_ + 0.54*Y*_Cl_ for the 26 solvents is shown in Figure 5. The points for TFE-H_2_O and HFIP-H_2_O are not included in the correlation but they are added to the plot to show the extent of their deviation from the line of best fit. In these highly ionizing aqueous fluoroalcohols, we propose as the dominant mechanism an ionization (S_N_1) process which involves rear-side nucleophilic solvation.

In the identical 26 solvents, the two-term G-W (2) analysis for PhOCOCl (**3**) yields  *R* = 0.949, *l* = 1.99 ± 0.16, *m* = 0.59 ± 0.04, *c* = 0.28 ± 0.07, and *F*-test = 104. The *l*/*m* ratio = 3.37 observed for** 3** is higher than the *l*/*m* ratio of 3.04 obtained above for** 2**.

There are four solvents, 100% EtOH, 80% EtOH, 100% MeOH, and 50% TFE, in which solvolyses of both** 2** [40] and** 3** [6, 11] are studied at the same temperature (25.0°C). A direct comparison of the specific rates of reaction for these solvents shows that PhOCOCl (**3**) is 19 times faster than FMOC-Cl (**2**) in 100% EtOH, 13 times faster in 80% EtOH, 20 times faster in 100% MeOH, and 4 times faster in 50% TFE. These ratios being slightly larger in the more nucleophilic solvents are consistent with the *l*/*m* ratio (3.37) being slightly larger than that of** 2** (3.04).

Additionally, the 3D image for FMOC-Cl (**2**′) shown in Figure 1 shows that the fluorenyl ring is forced out of the plane of the ether oxygen and that the ring is far removed from the carbonyl reaction center. As a result, any potential inductive or mesomeric effects exerted by the fluorenyl ring (through resonance) would be very weak with little influence on the rates of reaction of** 2**.

## 3. Conclusions

For 4,5-dimethoxy-2-nitrobenzyl chloroformate (NVOC-Cl,** 1**) a very good correlation was obtained from the use of the extended Grunwald-Winstein equation. The resultant *l*/*m* ratio of 2.85 is close to the *l*/*m* ratio of 2.95 obtained for phenyl chloroformate (PhOCOCl,** 3**) in an identical set of solvents. These values suggest a similarity of transition-state structures for the two compounds and an addition-elimination (A-E) process with a rate-determining addition step is proposed for** 1**.

The 3D image for NVOC-Cl (**1**′) shows that the two ether oxygens, the nitro group, and the aromatic ring are all in the same plane. Consequently relative to other benzylic substrates (PNZ-Cl and CBZ-Cl) a strong inductive effect is present in** 1**, and it solvolyzes at a much faster rate in solvents where the carbonyl-addition-elimination mechanism (A-E) is believed to be dominant (*k*_1_ > *k*_PNZ-Cl_ > *k*_CBZ-Cl_).

The exclusion of the rate data in the seven aqueous fluoroalcohols for solvolyses of** 2** leads to much improved correlations using the two-term Grunwald-Winstein equation. The *l*/*m* ratio of 3.04 and the significantly improved correlation observed in the log⁡(*k*/*k*_*o*_)_2_ versus log⁡(*k*/*k*_*o*_)_3_ regression plot are a strong indication that a two-step carbonyl-addition (A-E) process is occurring in the remaining 26 solvents. An ionization (S_N_1) process probably accompanied by rear-side solvation is proposed for** 2** in the seven TFE-H_2_O and HFIP-H_2_O mixtures.

A 3D image of 9-fluorenylmethyl chloroformate (FMOC-Cl,** 2**′) shows that the fluorenyl ring is twisted out of the plane containing the ether oxygen. This reduces any inductive or mesomeric effect and hence in the four common solvents studied at 25.0°C, the PhOCOCl substrate was found to solvolyze at a rate that was 4 to 20 times faster than** 2**.

## 4. Experimental Section

The 4,5-dimethoxy-2-nitrobenzyl chloroformate (NVOC-Cl, 97%, Sigma-Aldrich) was used as received. An approximately 1 M stock solution containing NVOC-Cl (**1**) in acetonitrile (99.8%, Sigma-Aldrich) was first made and a substrate concentration of at least 0.005 M in a variety of binary solvents was used in all of the experiments. All of the organic solvents were commercially available and they were purified using methods described previously [6]. The kinetic runs in constant temperature water baths were followed after sampling, using the titrimetric method. The specific rates and associated standard deviations, as presented in Table 1, were obtained by averaging all of the values from, at least, duplicate runs.

Multiple regression analyses were carried out using the Excel 2010 package from the Microsoft Corporation [41]. The 3D images presented in Figure 1 were computed using the KnowItAll Informatics System [42]. The KnowItAll platform contains a 3D molecular rendering program SymApps that uses a modified MM2 force field minimization module to convert 2D structure drawings to 3D images [42].

## Figures and Tables

**Table 1:** Specific rates of solvolysis (*k*) of **1** in several binary solvents at 25.0°C and literature values for *N*_T_ and *Y*_Cl_.

Solvent (%)^a^	**1** @ 25.0°C; 10^5^ *k, *s^−1^ ^b^	*N*_T_^c^	*Y*_Cl_^d^
100% EtOH	73.6 ± 1.2	0.37	−2.50
90% EtOH	116 ± 5	0.16	−0.90
80% EtOH	147 ± 10	0.00	0.00
100% MeOH	125 ± 10	0.17	−1.2
90% MeOH	187 ± 22	−0.01	−0.20
80% MeOH	299 ± 14	−0.06	0.67
90% acetone	3.23 ± 0.15	−0.35	−2.39
80% acetone	11.6 ± 0.6	−0.37	−0.80
70% acetone	23.3 ± 0.2	−0.42	0.17
97% TFE (w/w)	0.056 ± 0.003	−3.30	2.83
90% TFE (w/w)	0.346 ± 0.025	−2.55	2.85
70% TFE (w/w)	3.39 ± 0.24	−1.98	2.96
50% TFE (w/w)	16.9 ± 0.7	−1.73	3.16
60T-40E	5.77 ± 0.23	−0.94	0.63
40T-60E	17.2 ± 1.0	−0.34	0.48
20T-40E	34.0 ± 2.0	0.08	−1.42
90% HFIP (w/w)	0.065 ± 0.002	−3.84	4.31
80% HFIP (w/w)	0.117 ± 0.021	−3.31	3.99
70% HFIP (w/w)	3.61 ± 0.10	−2.94	3.83
50% HFIP (w/w)	9.71 ± 0.22	−2.49	3.80

^a^Substrate concentration of ca. 0.005 M; binary solvents on a volume-volume basis at 25.0°C, except for TFE-H_2_O and HFIP-H_2_O solvents which are on a weight-weight basis. T-E are TFE-ethanol mixtures. ^b^With associated standard deviation. ^c^References [16, 17]. ^d^References [18–20].

**Table 2:** Correlations of the specific rates of solvolysis of **1–3**, CBZ-Cl, and PNZ-Cl using the extended Grunwald-Winstein equation (2).

Substrate	*n* ^a^	*l* ^b^	*m* ^b^	*c* ^b^	*R* ^c^	*F* ^d^	*l*/*m*	Mechanism
**1**	20	1.48 ± 0.13	0.52 ± 0.08	0.04 ± 0.11	0.966	119	2.85	A-E
2^e,f^	26	1.64 ± 0.04	0.54 ± 0.04	0.13 ± 0.06	0.954	116	3.04	A-E
3^g^	49	1.66 ± 0.05	0.56 ± 0.03	0.15 ± 0.07	0.980	568	2.96	A-E
CBZ-Cl^h^	15	1.95 ± 0.16	0.57 ± 0.05	0.16 ± 0.15	0.966	83	3.42	A-E
	11	0.25 ± 0.05	0.66 ± 0.06	−2.05 ± 0.11	0.976	80	0.38	Ionization-fragmentation
PNZ-Cl^i^	19	1.61 ± 0.09	0.46 ± 0.04	0.04 ± 0.22	0.975	157	3.50	A-E

^a^ *n* is the number of solvents. ^b^With associated standard error. ^c^Correlation coefficient. ^d^ *F*-test value. ^e^Values taken from [40]. ^f^Excluding the data points in aqueous HFIP and aqueous TFE, in regression calculations. Values taken from [40]. ^g^Correlation data from [11]. ^h^Correlation data from [37]. ^i^Correlation data from [37, 38].
